# (*N*→*B*)-4-Methyl-3-pyrid­yl[*N*-methyl­imino­diacetate-*O*,*O*′,*N*]borane

**DOI:** 10.1107/S1600536812040974

**Published:** 2012-10-06

**Authors:** Marek Dąbrowski, Krzysztof Durka, Janusz Serwatowski

**Affiliations:** a Physical Chemistry Department, Faculty of Chemistry, Warsaw University of, Technology, Noakowskiego 3, 00-664 Warsaw, Poland

## Abstract

The title compound, C_11_H_13_BN_2_O_4_, has a rigid bicyclic structure due to an intra­molecular nitro­gen–boron dative bond. The B atom is in a distorted tetra­hedron environment with a B—N bond length of 1.640 (2) Å, which is in good comparison with the values in analogues compounds. In the crystal, the mol­ecules are linked by weak C—H⋯O and C—H⋯N inter­actions, forming a three-dimensional network.

## Related literature

For related structures of [*N*-alkyl­imino­diacetate-*O*,*O′*,*N*]boranes, see: Mancilla *et al.* (1997 ▶, 2005 ▶); Gillis & Burke (2008 ▶); Knapp *et al.* (2009 ▶); Percino *et al.* (2009 ▶).
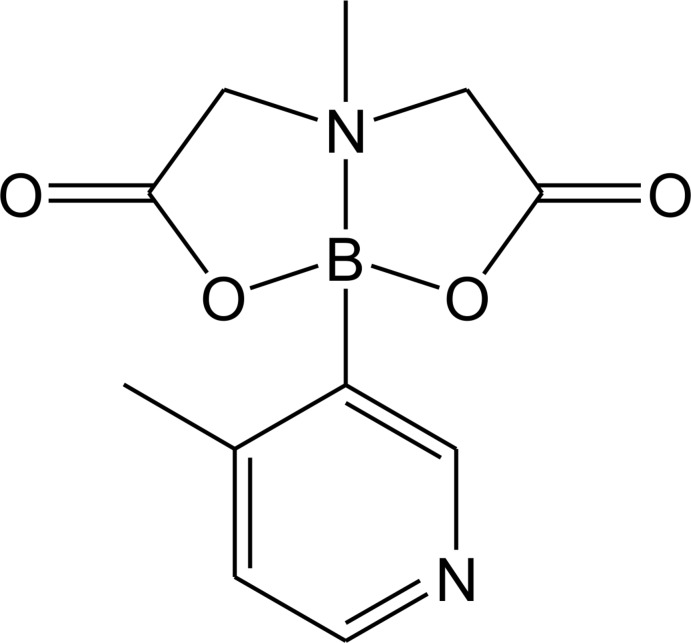


## Experimental

### 

#### Crystal data

C_11_H_13_BN_2_O_4_
*M* *_r_* = 248.04
Monoclinic, 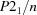

*a* = 7.306 (3) Å
*b* = 14.7425 (4) Å
*c* = 10.8281 (19) Å
β = 96.91 (4)°
*V* = 1157.8 (5) Å^3^
*Z* = 4
Mo *K*α radiation
μ = 0.11 mm^−1^
*T* = 100 K
0.16 × 0.12 × 0.10 mm

#### Data collection

Agilent Xcalibur Opal diffractometer
Absorption correction: multi-scan (*CrysAlis PRO*; Agilent, 2011 ▶) *T*_min_ = 0.910, *T*_max_ = 0.989
26273 measured reflections
3993 independent reflections
3148 reflections with *I* > 2σ(*I*)
*R*_int_ = 0.075

#### Refinement

*R*[*F* ^2^ > 2σ(*F* ^2^)] = 0.052
*wR*(*F* ^2^) = 0.147
*S* = 1.07
3993 reflections
163 parameters
H-atom parameters constrained
Δρ_max_ = 0.47 e Å^−3^
Δρ_min_ = −0.43 e Å^−3^

### 

Data collection: *CrysAlis PRO* (Agilent, 2011 ▶); cell refinement: *CrysAlis PRO*; data reduction: *CrysAlis PRO*; program(s) used to solve structure: *SHELXTL* (Sheldrick, 2008 ▶); program(s) used to refine structure: *SHELXTL*; molecular graphics: *DIAMOND* (Brandenburg, 2005 ▶); software used to prepare material for publication: *SHELXTL* and *PLATON* (Spek, 2009 ▶).

## Supplementary Material

Crystal structure: contains datablock(s) global, I. DOI: 10.1107/S1600536812040974/is5196sup1.cif

Structure factors: contains datablock(s) I. DOI: 10.1107/S1600536812040974/is5196Isup2.hkl

Additional supplementary materials:  crystallographic information; 3D view; checkCIF report

## Figures and Tables

**Table 1:** Hydrogen-bond geometry (Å, °)

*D*—H⋯*A*	*D*—H	H⋯*A*	*D*⋯*A*	*D*—H⋯*A*
C8—H10*A*⋯O4^i^	0.99	2.42	3.356 (1)	158
C11—H12*B*⋯N1^ii^	0.98	2.64	3.509 (1)	149
C11—H12*A*⋯O4^i^	0.98	2.40	3.259 (1)	146
C10—H8*A*⋯O3^iii^	0.99	2.28	3.247 (1)	165
C11—H12*C*⋯O2^iv^	0.98	2.57	3.500 (1)	158

Symmetry codes: (i) 
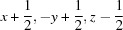
; (ii) 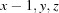; (iii) 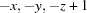; (iv) 
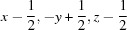
.

**Fig. 1.**
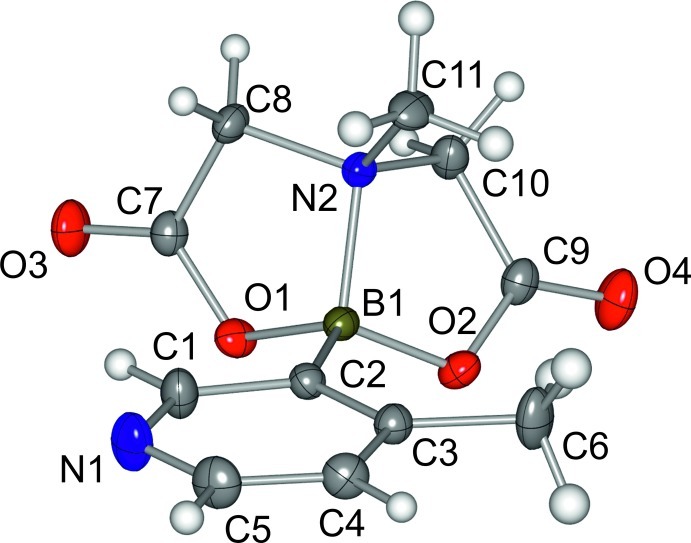
The molecular structure of the title compound with the atom numbering scheme. Displacement ellipsoids for non-H atoms are drawn at the 50% probability level.

**Fig. 2.**
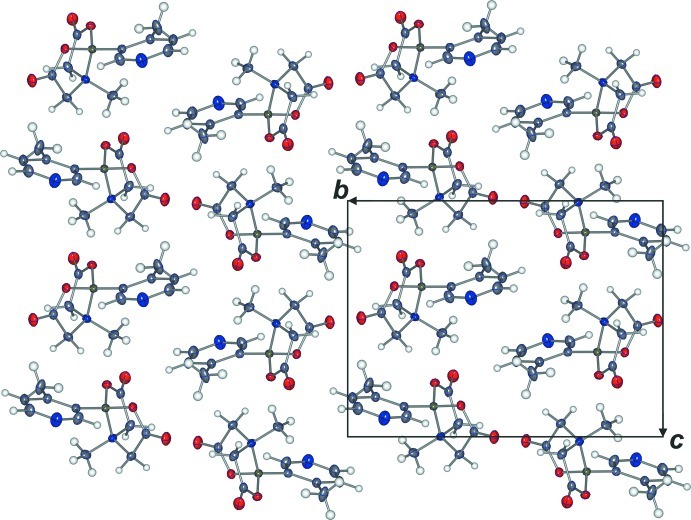
A packing diagram of the title compound, viewed along the a axis.

## supplementary crystallographic information

### Comment

Boron heterocycles derived from amino acids have received considerable attention due to their utilization in organic synthesis and medicine. Boronic acids that are susceptible to degradations are protected with *N*-methyliminodiacetic acid (MIDA) ligand. The MIDA boronates are compatible with a wide range of common synthetic reagents, allowing them to be functionalized to create complex boronic acid derivatives (Mancilla *et al.*, 1997, 2005; Gillis *et al.*, 2008; Knapp *et al.*, 2009; Percino *et al.*, 2009). Our interest has focused on the MIDA esters of pyridineboronic acids and their structural behavior.

In the title compound, the boron atom has a tetrahedral geometry with bond angles at the B atom ranging from 99.1 (2) to 116.8 (1)°. The B—O [1.466 (2) and 1.489 (2) Å] and B—C [1.595 (2) Å] bond lengths are in the normal ranges for such compounds. The B—N bond length is equal to 1.640 (2) Å and is similar to the values found in the other structures of MIDA boronate esters. As indicated by the C1—B1—N2—C11 and C2—C2—B1—O1 torsion angles, the bicycle ring is significantly distorted and the aryl unit is twisted along C—B bond. The crystal structure is dominated by weak C—H···O and C—H···N hydrogen interactions (Table 1).

### Experimental

The title compound was received from Aldrich. Single crystals suitable for X-ray diffraction analysis were grown by cooling a solution of the ester (0.2 g) in diethyl ether (10 ml) and dimethyl sulfoxide (5 ml).

### Refinement

All H atoms were placed in calculated positions with C—H distances of 0.95 Å (phenyl), 0.98 Å (methyl) and 0.99 Å (methylene). They were located in difference maps and were included in the refinement in riding approximation with *U*_iso_(phenyl and methylene H) = 1.2*U*_eq_(C) and *U*_iso_(methyl H) = 1.5*U*_eq_(C).

### Crystal data

**Table:**

C_11_H_13_BN_2_O_4_	*F*(000) = 520
*M**_r_* = 248.04	*D*_x_ = 1.423 Mg m^−^^3^
Monoclinic, *P*2_1_/*n*	Mo *K*α radiation, λ = 0.7107 Å
Hall symbol: -P 2yn	Cell parameters from 9561 reflections
*a* = 7.306 (3) Å	θ = 1.9–32.2°
*b* = 14.7425 (4) Å	µ = 0.11 mm^−^^1^
*c* = 10.8281 (19) Å	*T* = 100 K
β = 96.91 (4)°	Unshaped, colourless
*V* = 1157.8 (5) Å^3^	0.16 × 0.12 × 0.10 mm
*Z* = 4	

### Data collection

**Table:**

Agilent Xcalibur Opal diffractometer	3993 independent reflections
Radiation source: Enhance (Mo) X-ray Source	3148 reflections with *I* > 2σ(*I*)
Graphite monochromator	*R*_int_ = 0.075
Detector resolution: 8.4441 pixels mm^-1^	θ_max_ = 32.3°, θ_min_ = 2.3°
ω scan	*h* = −10→10
Absorption correction: multi-scan (*CrysAlis PRO*; Agilent, 2011)	*k* = −21→21
*T*_min_ = 0.910, *T*_max_ = 0.989	*l* = −16→16
26273 measured reflections	

### Refinement

**Table:**

Refinement on *F*^2^	Primary atom site location: structure-invariant direct methods
Least-squares matrix: full	Secondary atom site location: difference Fourier map
*R*[*F*^2^ > 2σ(*F*^2^)] = 0.052	Hydrogen site location: inferred from neighbouring sites
*wR*(*F*^2^) = 0.147	H-atom parameters constrained
*S* = 1.07	*w* = 1/[σ^2^(*F*_o_^2^) + (0.0715*P*)^2^ + 0.4349*P*] where *P* = (*F*_o_^2^ + 2*F*_c_^2^)/3
3993 reflections	(Δ/σ)_max_ < 0.001
163 parameters	Δρ_max_ = 0.47 e Å^−^^3^
0 restraints	Δρ_min_ = −0.43 e Å^−^^3^

### Special details

**Table:**

Geometry. All e.s.d.'s (except the e.s.d. in the dihedral angle between two l.s. planes) are estimated using the full covariance matrix. The cell e.s.d.'s are taken into account individually in the estimation of e.s.d.'s in distances, angles and torsion angles; correlations between e.s.d.'s in cell parameters are only used when they are defined by crystal symmetry. An approximate (isotropic) treatment of cell e.s.d.'s is used for estimating e.s.d.'s involving l.s. planes.
Refinement. Refinement of *F*^2^ against ALL reflections. The weighted *R*-factor *wR* and goodness of fit *S* are based on *F*^2^, conventional *R*-factors *R* are based on *F*, with *F* set to zero for negative *F*^2^. The threshold expression of *F*^2^ > σ(*F*^2^) is used only for calculating *R*-factors(gt) *etc*. and is not relevant to the choice of reflections for refinement. *R*-factors based on *F*^2^ are statistically about twice as large as those based on *F*, and *R*- factors based on ALL data will be even larger.

### Fractional atomic coordinates and isotropic or equivalent isotropic displacement parameters (Å^2^)

**Table:**

	*x*	*y*	*z*	*U*_iso_*/*U*_eq_	
C11	−0.2305 (2)	0.29350 (10)	0.44416 (12)	0.0241 (3)	
H12A	−0.1311	0.3273	0.4117	0.036*	
H12B	−0.2901	0.3322	0.5011	0.036*	
H12C	−0.3216	0.2748	0.3750	0.036*	
O1	0.10475 (13)	0.14926 (6)	0.64076 (9)	0.0195 (2)	
O2	−0.13312 (13)	0.22847 (6)	0.73583 (8)	0.0196 (2)	
N2	−0.15248 (15)	0.21153 (7)	0.51222 (9)	0.0157 (2)	
C2	0.11011 (17)	0.32272 (8)	0.63785 (11)	0.0167 (2)	
O3	0.20124 (15)	0.04826 (7)	0.50831 (11)	0.0275 (2)	
O4	−0.41001 (16)	0.17271 (7)	0.76432 (11)	0.0305 (3)	
C8	−0.04008 (19)	0.15473 (9)	0.43621 (12)	0.0198 (3)	
H10A	0.0208	0.1927	0.3777	0.024*	
H10B	−0.1177	0.1087	0.3882	0.024*	
C3	0.06232 (18)	0.40943 (9)	0.67863 (12)	0.0188 (2)	
C9	−0.29166 (19)	0.18660 (8)	0.69872 (12)	0.0199 (3)	
B1	−0.01072 (19)	0.23263 (9)	0.63818 (12)	0.0157 (2)	
C10	−0.30028 (19)	0.15837 (9)	0.56364 (13)	0.0213 (3)	
H8A	−0.2776	0.0925	0.5570	0.026*	
H8B	−0.4226	0.1727	0.5181	0.026*	
C4	0.1896 (2)	0.47963 (9)	0.67544 (13)	0.0228 (3)	
H4	0.1618	0.5384	0.7041	0.027*	
C7	0.10090 (18)	0.11005 (8)	0.53041 (12)	0.0189 (2)	
C1	0.28286 (19)	0.31591 (10)	0.59509 (14)	0.0246 (3)	
H1	0.3165	0.2579	0.5669	0.029*	
N1	0.40557 (18)	0.38297 (9)	0.58999 (14)	0.0305 (3)	
C6	−0.1176 (2)	0.43051 (10)	0.72727 (17)	0.0310 (3)	
H6A	−0.1941	0.3757	0.7236	0.046*	
H6B	−0.1825	0.4781	0.6762	0.046*	
H6C	−0.0934	0.4514	0.8136	0.046*	
C5	0.3558 (2)	0.46393 (10)	0.63083 (15)	0.0274 (3)	
H5	0.4393	0.5132	0.6290	0.033*	

### Atomic displacement parameters (Å^2^)

**Table:**

	*U*^11^	*U*^22^	*U*^33^	*U*^12^	*U*^13^	*U*^23^
C11	0.0284 (7)	0.0246 (6)	0.0183 (6)	0.0100 (5)	−0.0018 (5)	0.0020 (5)
O1	0.0217 (5)	0.0160 (4)	0.0201 (4)	0.0042 (3)	−0.0009 (3)	0.0005 (3)
O2	0.0235 (5)	0.0203 (4)	0.0153 (4)	−0.0028 (3)	0.0043 (3)	−0.0011 (3)
N2	0.0161 (5)	0.0157 (4)	0.0153 (4)	0.0022 (4)	0.0014 (4)	−0.0016 (3)
C2	0.0160 (5)	0.0167 (5)	0.0173 (5)	0.0009 (4)	0.0016 (4)	−0.0001 (4)
O3	0.0270 (5)	0.0197 (5)	0.0372 (6)	0.0076 (4)	0.0092 (4)	−0.0022 (4)
O4	0.0326 (6)	0.0271 (5)	0.0355 (6)	−0.0047 (4)	0.0198 (5)	−0.0006 (4)
C8	0.0228 (6)	0.0207 (6)	0.0164 (5)	0.0042 (5)	0.0043 (4)	−0.0027 (4)
C3	0.0192 (6)	0.0171 (5)	0.0201 (5)	0.0006 (4)	0.0030 (4)	−0.0009 (4)
C9	0.0227 (6)	0.0147 (5)	0.0234 (6)	0.0006 (4)	0.0080 (5)	−0.0012 (4)
B1	0.0172 (6)	0.0152 (6)	0.0144 (5)	0.0016 (5)	0.0010 (5)	−0.0007 (4)
C10	0.0186 (6)	0.0204 (6)	0.0257 (6)	−0.0026 (5)	0.0052 (5)	−0.0057 (5)
C4	0.0261 (7)	0.0155 (5)	0.0268 (6)	−0.0019 (5)	0.0030 (5)	0.0003 (5)
C7	0.0195 (6)	0.0148 (5)	0.0231 (6)	0.0007 (4)	0.0053 (5)	0.0001 (4)
C1	0.0192 (6)	0.0231 (6)	0.0325 (7)	−0.0002 (5)	0.0079 (5)	−0.0049 (5)
N1	0.0219 (6)	0.0286 (6)	0.0431 (7)	−0.0049 (5)	0.0120 (5)	−0.0048 (5)
C6	0.0267 (7)	0.0213 (6)	0.0475 (9)	0.0007 (5)	0.0155 (7)	−0.0108 (6)
C5	0.0254 (7)	0.0232 (6)	0.0344 (7)	−0.0073 (5)	0.0062 (6)	0.0010 (5)

### Geometric parameters (Å, º)

**Table:**

C11—N2	1.4924 (17)	C8—H10A	0.9900
C11—H12A	0.9800	C8—H10B	0.9900
C11—H12B	0.9800	C3—C4	1.3947 (18)
C11—H12C	0.9800	C3—C6	1.506 (2)
O1—C7	1.3246 (16)	C9—C10	1.5147 (19)
O1—B1	1.4891 (16)	C10—H8A	0.9900
O2—C9	1.3313 (17)	C10—H8B	0.9900
O2—B1	1.4660 (17)	C4—C5	1.379 (2)
N2—C8	1.4884 (16)	C4—H4	0.9500
N2—C10	1.4950 (18)	C1—N1	1.3403 (19)
N2—B1	1.6400 (19)	C1—H1	0.9500
C2—C1	1.3993 (19)	N1—C5	1.338 (2)
C2—C3	1.4101 (17)	C6—H6A	0.9800
C2—B1	1.5950 (19)	C6—H6B	0.9800
O3—C7	1.2111 (16)	C6—H6C	0.9800
O4—C9	1.2009 (17)	C5—H5	0.9500
C8—C7	1.511 (2)		
			
N2—C11—H12A	109.5	O2—B1—C2	114.98 (10)
N2—C11—H12B	109.5	O1—B1—C2	112.01 (11)
H12A—C11—H12B	109.5	O2—B1—N2	102.29 (10)
N2—C11—H12C	109.5	O1—B1—N2	99.13 (9)
H12A—C11—H12C	109.5	C2—B1—N2	116.77 (10)
H12B—C11—H12C	109.5	N2—C10—C9	105.53 (10)
C7—O1—B1	113.13 (10)	N2—C10—H8A	110.6
C9—O2—B1	112.70 (10)	C9—C10—H8A	110.6
C8—N2—C11	112.70 (10)	N2—C10—H8B	110.6
C8—N2—C10	112.48 (10)	C9—C10—H8B	110.6
C11—N2—C10	110.99 (11)	H8A—C10—H8B	108.8
C8—N2—B1	103.37 (10)	C5—C4—C3	120.13 (13)
C11—N2—B1	115.00 (10)	C5—C4—H4	119.9
C10—N2—B1	101.65 (9)	C3—C4—H4	119.9
C1—C2—C3	115.84 (12)	O3—C7—O1	123.98 (13)
C1—C2—B1	117.55 (11)	O3—C7—C8	125.05 (12)
C3—C2—B1	126.60 (11)	O1—C7—C8	110.94 (11)
N2—C8—C7	104.40 (10)	N1—C1—C2	126.53 (13)
N2—C8—H10A	110.9	N1—C1—H1	116.7
C7—C8—H10A	110.9	C2—C1—H1	116.7
N2—C8—H10B	110.9	C5—N1—C1	115.76 (13)
C7—C8—H10B	110.9	C3—C6—H6A	109.5
H10A—C8—H10B	108.9	C3—C6—H6B	109.5
C4—C3—C2	118.29 (12)	H6A—C6—H6B	109.5
C4—C3—C6	117.93 (12)	C3—C6—H6C	109.5
C2—C3—C6	123.78 (12)	H6A—C6—H6C	109.5
O4—C9—O2	124.26 (13)	H6B—C6—H6C	109.5
O4—C9—C10	125.11 (13)	N1—C5—C4	123.44 (13)
O2—C9—C10	110.63 (11)	N1—C5—H5	118.3
O2—B1—O1	110.20 (10)	C4—C5—H5	118.3
			
C11—N2—C8—C7	150.72 (11)	C10—N2—B1—O2	−25.44 (11)
C10—N2—C8—C7	−82.89 (13)	C8—N2—B1—O1	−29.06 (11)
B1—N2—C8—C7	25.95 (12)	C11—N2—B1—O1	−152.32 (10)
C1—C2—C3—C4	−1.18 (19)	C10—N2—B1—O1	87.71 (11)
B1—C2—C3—C4	177.63 (12)	C8—N2—B1—C2	91.36 (12)
C1—C2—C3—C6	179.70 (14)	C11—N2—B1—C2	−31.90 (15)
B1—C2—C3—C6	−1.5 (2)	C10—N2—B1—C2	−151.87 (11)
B1—O2—C9—O4	177.44 (13)	C8—N2—C10—C9	134.54 (11)
B1—O2—C9—C10	−1.95 (15)	C11—N2—C10—C9	−98.15 (12)
C9—O2—B1—O1	−87.36 (13)	B1—N2—C10—C9	24.61 (12)
C9—O2—B1—C2	144.91 (11)	O4—C9—C10—N2	164.50 (13)
C9—O2—B1—N2	17.32 (13)	O2—C9—C10—N2	−16.11 (14)
C7—O1—B1—O2	129.43 (11)	C2—C3—C4—C5	1.3 (2)
C7—O1—B1—C2	−101.22 (12)	C6—C3—C4—C5	−179.51 (14)
C7—O1—B1—N2	22.64 (13)	B1—O1—C7—O3	170.72 (12)
C1—C2—B1—O2	149.59 (12)	B1—O1—C7—C8	−7.42 (15)
C3—C2—B1—O2	−29.19 (18)	N2—C8—C7—O3	168.47 (12)
C1—C2—B1—O1	22.78 (16)	N2—C8—C7—O1	−13.41 (14)
C3—C2—B1—O1	−156.01 (12)	C3—C2—C1—N1	0.4 (2)
C1—C2—B1—N2	−90.54 (14)	B1—C2—C1—N1	−178.55 (14)
C3—C2—B1—N2	90.67 (15)	C2—C1—N1—C5	0.4 (2)
C8—N2—B1—O2	−142.20 (10)	C1—N1—C5—C4	−0.2 (2)
C11—N2—B1—O2	94.54 (12)	C3—C4—C5—N1	−0.6 (2)

### Hydrogen-bond geometry (Å, º)

**Table:**

*D*—H···*A*	*D*—H	H···*A*	*D*···*A*	*D*—H···*A*
C8—H10*A*···O4^i^	0.99	2.42	3.356 (1)	158
C11—H12*B*···N1^ii^	0.98	2.64	3.509 (1)	149
C11—H12*A*···O4^i^	0.98	2.40	3.259 (1)	146
C10—H8*A*···O3^iii^	0.99	2.28	3.247 (1)	165
C11—H12*C*···O2^iv^	0.98	2.57	3.500 (1)	158

Symmetry codes: (i) *x*+1/2, −*y*+1/2, *z*−1/2; (ii) *x*−1, *y*, *z*; (iii) −*x*, −*y*, −*z*+1; (iv) *x*−1/2, −*y*+1/2, *z*−1/2.
